# The Risk of Growth Disturbance Is Low After Pediatric Anterior Cruciate Ligament Reconstruction With a Femoral Growth Plate Sparing Technique

**DOI:** 10.1016/j.asmr.2023.100793

**Published:** 2023-09-29

**Authors:** Peter Ziegler Faunø, Jannie Bøge Steinmeier Larsen, Mette Mølby Nielsen, Michel Hellfritzsch, Torsten Grønbech Nielsen, Martin Lind

**Affiliations:** Department of Sports Medicine, Aarhus University Hospital, Aarhus, Denmark

## Abstract

**Purpose:**

To evaluate radiological tibial and femoral length and axis growth disturbances, as well as clinical outcome in skeletal immature anterior cruciate ligament reconstruction (ACLR) patients treated with a femoral growth plate-sparing ACLR technique.

**Methods:**

Skeletally immature patients who underwent operation between 2013 to 2019 with ALCR using the femoral growth plate-sparing technique were investigated with follow-up after growth plate closure. The inclusion criteria were isolated ACL rupture in patients with open physis in the distal femur and proximal tibia seen at plain radiography. The minimum follow-up time was 29 months. Patients were evaluated with full extremity radiographs measuring limb length discrepancy and coronal knee alignment compared to contralateral leg, as well as clinical evaluation with Rolimeter measurements and the Knee Osteoarthritis Outcome Score (KOOS), the International Knee Documentation Committee subjective knee form (IKDC), and Tegner Activity Scale scores.

**Results:**

Sixty-five patients were examined with radiography, and 52 patients were assessed with clinical examination**.** The mean follow-up time was 68 (range, 29-148) months. No limb-length discrepancy (−0.65 mm [confidence interval {CI}, −2.21 to 0.92]) or angular deformity at tibia (−0.25° [CI, −0.78° to 0.28°]) was found. There was a small but statistically significant different angular deformity at the distal femur compared to the contralateral leg (−1.51° [CI, −2.31 to −0.72]) at follow-up. The side-to-side difference in knee laxity at follow-up was 2.4 mm. At follow-up the KOOS Sport, KOOS Quality of Life (QoL), IKDC, and Tegner scores were 80, 75, 86, and 5, respectively. Sixty-seven percent of the patients met the Patient Acceptable Symptom State, and 52% reported results exceeding the KOOS Sport MCID Level and 69% the KOOS QoL level.

**Conclusions:**

Femoral physis-sparing ALCR is associated with a low risk of alignment and length disturbances. The technique provides otherwise good subjective clinical outcome and knee stability.

**Level of Evidence:**

Level IV, therapeutic case series.

The incidence of anterior cruciate ligament (ACL) injuries among children is substantial and has increased rapidly over recent years.^1^, ^2^, ^3^, ^4^ According to the Danish ACL register, 6% of all ACL reconstructions (ALCR) are performed in patients under 15 years of age.^5^

One of the main controversies regarding optimal treatment of the ACL-injured child is the concern about surgically-induced physeal growth disturbance. Nonoperative treatment may not sufficiently prevent knee instability and can lead to further meniscal and articular cartilage damage because of recurrent instability episodes.^6^^,^^7^ Operative treatment, on the other hand, may cause growth disturbances of the involved leg because of surgical injury to the physes.^8^, ^9^, ^10^

Any postoperative change in the knee might lead to increased risk of arthrosis.^11^ Valgus knee has also been associated with an increased risk of ACL injury in adults.^12^ Little is known with regard to increased valgus after pediatric ACLR. However, the literature regarding outcomes after surgical treatment of ACL injury in children demonstrates good clinical results comparable to ALCR in adults.^13^

The purpose of this study was to quantify the degree of growth disturbances in the operated limb compared to the nonoperated limb using a hybrid ALCR technique with all epiphyseal femoral drilling and transphyseal tibial drilling. Furthermore, the subjective and objective clinical outcomes were analyzed. We hypothesized that using the femoral growth plate-sparing technique would result in a low incidence of growth disturbances.

## Methods

Skeletally immature patients who underwent ACLR by a single surgeon (P.F.) between 2013 and 2019 were identified. Skeletal immaturity of the patients was documented by preoperative X-ray films clearly demonstrating an open epiphysis in the distal femur and proximal tibia. The indication for surgery was a wish to return to pivoting sport or failed conservative treatment.

The inclusion criteria were patients with isolated ACL rupture with open physis in the distal femur and proximal tibia in preoperative X-ray films. Exclusion criteria were multiligament knee injuries, revision cases, and injury to the other knee. At follow-up patients should be above age 18 and demonstrate closed physis around the knee at follow-up radiography.

### Surgery and Rehabilitation

The operations were done at 1 center by 1 experienced surgeon (P.F.). The indication for ALCR was a desire to return to pivoting sports after ACL rupture or failed conservative treatment.

The femoral tunnel was placed below the physis, whereas the tibial tunnel was placed transphyseally. Both tunnels were created with a retro-drill (Twister; DePuy, Warsaw, IN). They were both 25 mm long, and the diameter was adjusted according to the size of the graft.

**Fig 1:**
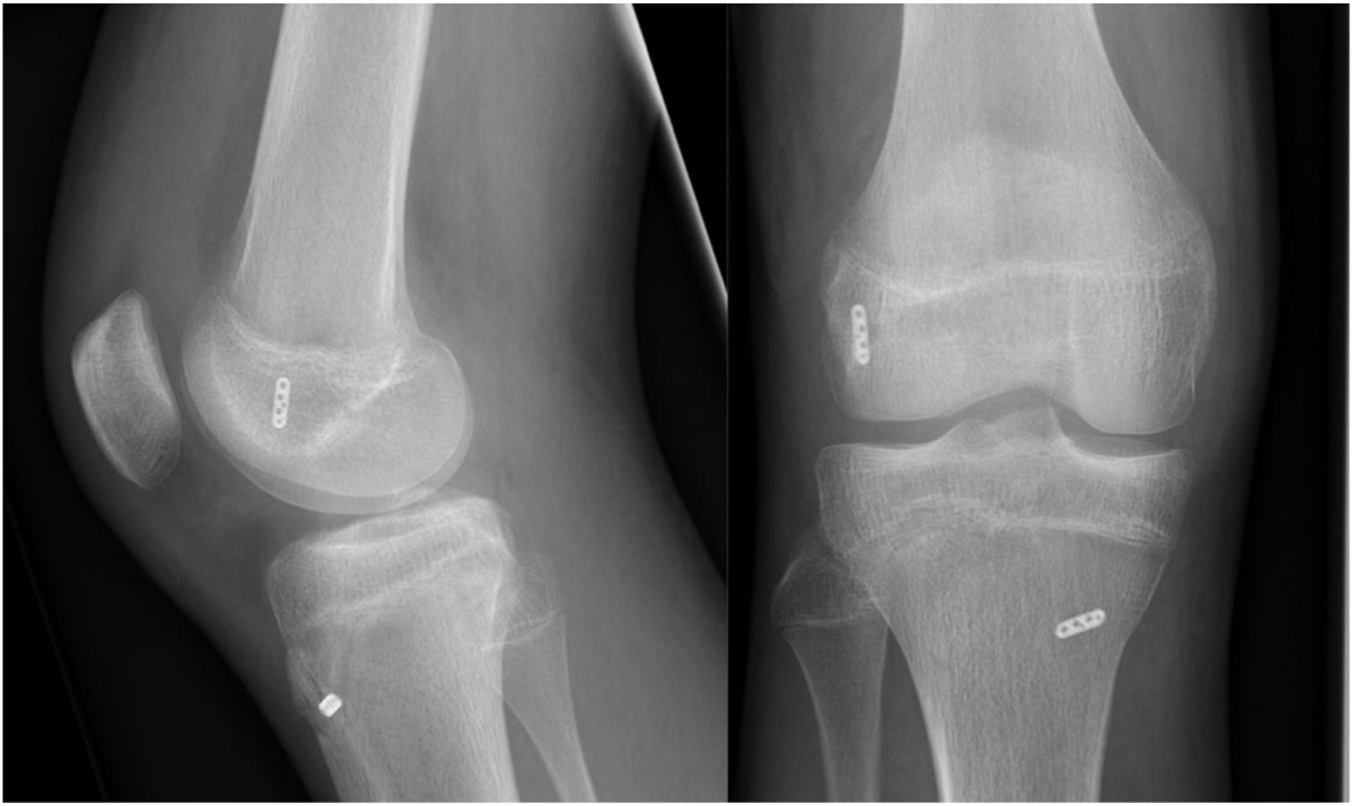
All operated patients underwent postoperative radiography in sagittal and frontal plane to confirm the correct position of the tunnels.

An ipsilateral harvested quadrupled semitendinosus autograft was secured in both ends with extracortical fixation, (Rigidloop; DePuy). The position of the tunnels was documented by radiography (Fig 1).

**Fig 2:**
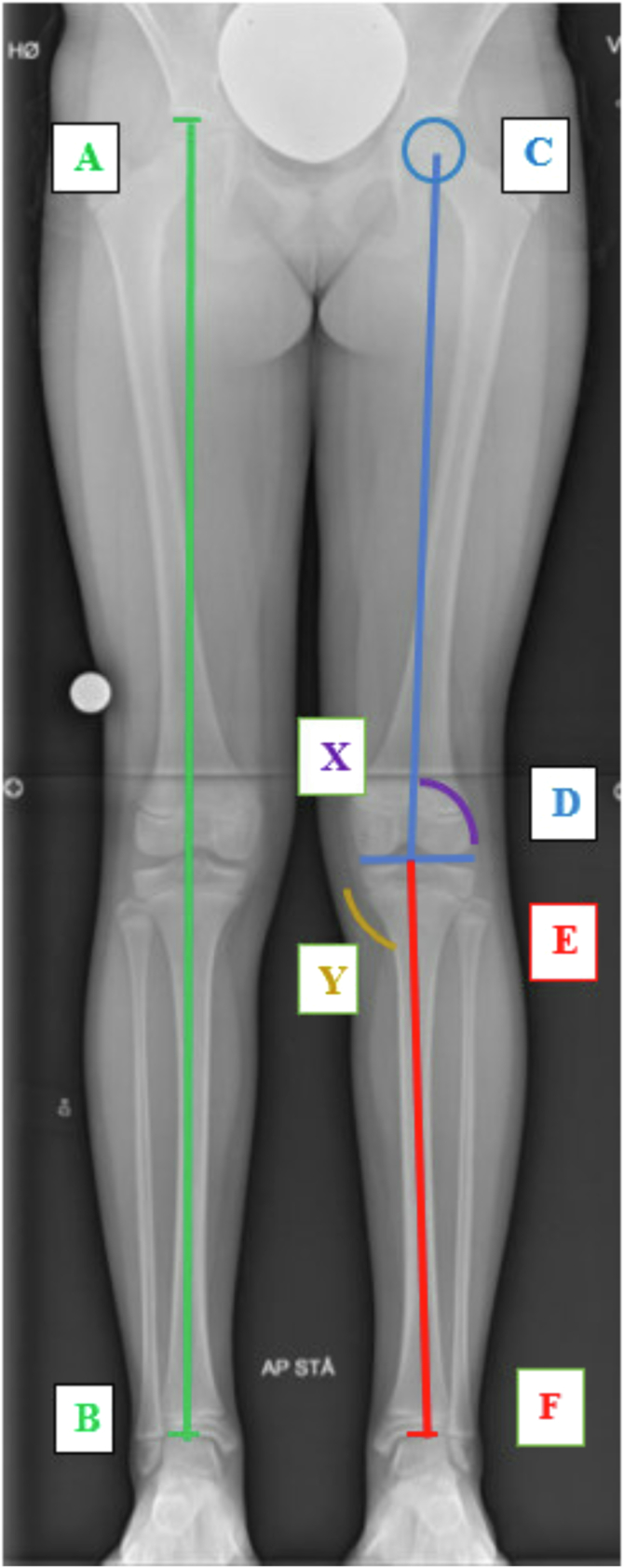
Radiological assessment of leg length and knee angulation compared to not injured knee. (A, B) Limb length measured from superior aspect of femoral head to tibial distal tibial joint line. (C, D) Femoral length measured from the superior aspect of the imaged femoral head and the distal portion of the medial femoral condyle. (E, F) Tibial length, measured from superior of tibial spine to distal tibia joint line X: mMDFA Mechanical Medial Distal Femur Angle. Y: mMPTA, mechanical medial proximal tibial angle.

The patients were allowed postoperative full weightbearing as tolerated, using crutches 2 weeks after surgery. Patients received physiotherapist-assisted rehabilitation for 5 to 11 months after surgery. It was stressed to all patients and their parents that participation in pivoting sports less than 1 year after surgery was associated with a high risk of graft rupture. Return to non-pivoting sport was allowed after 6 months and Pivoting sport was allowed after 12 months.

### Radiological Evaluation

At final follow-up patients were evaluated using full-extremity radiographs to assess leg length discrepancy and angular malalignment. Growth disturbance was evaluated at maturity with axial and coronal high precision radiographic analysis by comparison to the nonoperated lower extremity.

The radiological examinations were performed by 1 experienced radiologist using the Adora DRFi system (NRT X-RAY A/S, Hasselager, Denmark), with an optional application installed that was designed for measurement of lower limb geometry. A run of radiographic images used for leg measurements was obtained with the normal acquisition protocol with the patients in standing position with their back against the vertical tabletop and with full bodyweight on the leg displayed. The image dataset was transferred to a “Picture Archiving and Communication System” (Pacs). The algorithm for the leg composite image reconstruction was specifically designed for a series of images of the lower limb skeleton taking specific features of these images into account. The package reconstructed a single composite image from a run of X-ray images made for this purpose. The composite image was used for length of femur and tibia and angle measurements around the knee (Fig 2). The angle and length measurements were performed by defining specific anatomical landmarks in the composite image. Thereafter, the software program automatically calculated the measurements. An axial limb length discrepancy of more than 10 mm and a coronal plane ≥5° side-to side angle difference as clinically relevant were defined.

**Fig 3:**
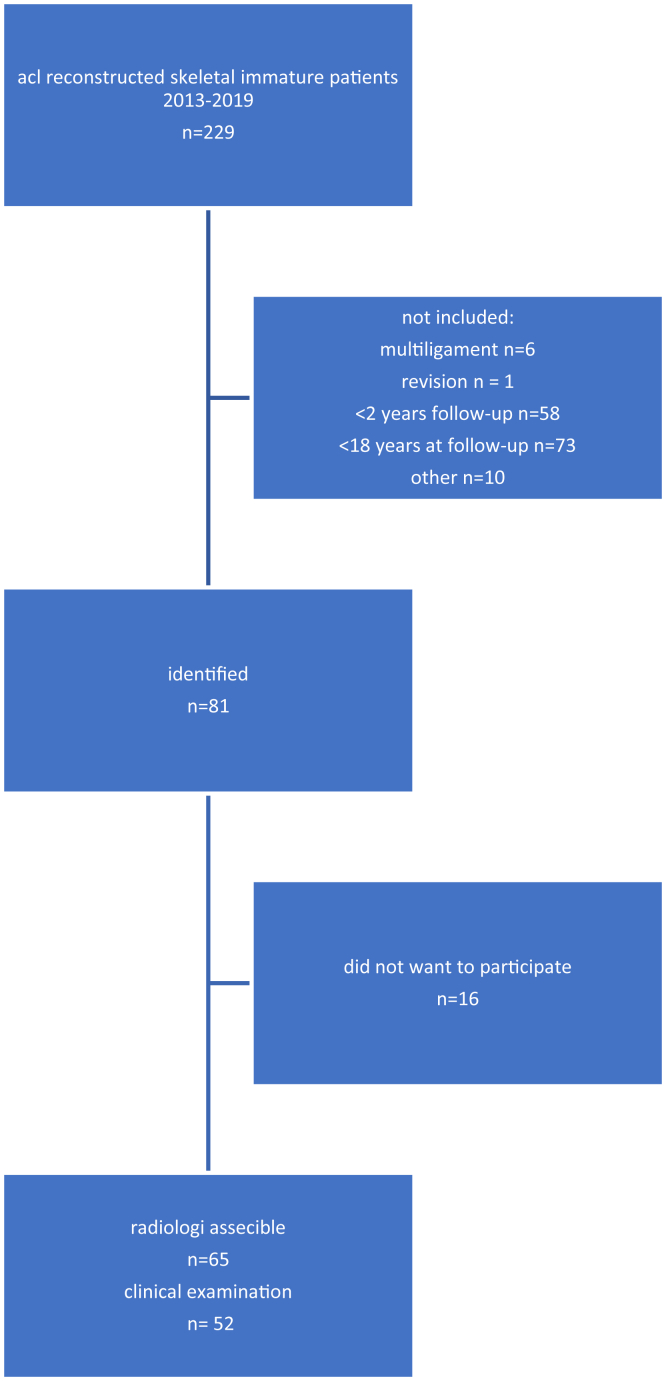
Flow diagram of included patients. In the period 2013–2019, 229 patients underwent operation with a femoral physis-sparing technique. Eighty-one patients met the inclusion criteria, of which 16 were unable or did not want to participate in the study. Fifty-two patients underwent clinical examination and radiology. A further 13 patients were not able to come to the clinical examination and were assessed with radiology only.

### Clinical Outcome Evaluation

A clinical outcome evaluation was performed using both a subjective evaluation and an objective evaluation. The subjective evaluation included the Knee Osteoarthritis Outcome Score (KOOS),^14^ the International Knee Documentation Committee subjective knee form (IKDC), and the Tegner Activity Scale (Tegner).^15^

The KOOS consists of 5 subscales: Symptoms, Pain, Activities of Daily Living (ADL), Sport/Recreation (Sport), and Quality of Life (QoL). Each subscale ranges from 0 to 100, with 0 representing extreme problems and 100 representing no problems at all. Only KOOS Sport and KOOS QoL are useful for ACL patients.^12^ Minimal clinically important difference (MCID) is calculated for KOOS Sport and QoL and the cohort values were 18.3 and 12.1 points, respectively.^16^ The Patient Acceptable Symptom State (PASS) thresholds for KOOS Sport and KOOS QoL are set to 75.0 points and 62.5 points, respectively.^17^

The IKDC score ranges from 0 to 100, with 0 representing extreme problems and 100 representing no problems at all. PASS scores are set to 75.9 points.^17^ The MCID for IKDC will not be calculated because the baseline IKDC was not collected.

The Tegner score range from 0 to 10, with 0 representing disability of function and 10 representing national or internal sports level. The MCID for Tegner was set to 1.0 point. PASS scores are not available for the Tegner score.

Objective knee laxity for all patients was evaluated by Rolimeter measurements performed by the same experienced physiotherapist. The side-to-side difference at maximum force was measured. The study was approved by the regional ethical committee (EC. 1-10-72-246-20).

### Statistical Analysis

Normal-distributed data were presented as mean (standard deviation [SD]), means (confidence interval [CI]) or else presented as medians (quartiles). Paired Student *t*-test and Wilcoxon rang sum test were used for normally and non-normally distributed data, respectively. Proportions were presented as number (n (%)), and data were compared using the χ^2^ analysis. *P* values <.05 were considered statically significant. Statistical analysis was computed using Stata version 17 (Stata Release 12, College Station, TX) and Excel version 2016.

## Results

A total of 229 pediatric patients who underwent ACLR during the study period were identified. Sixty-five patients with valid X-ray films were included in the study. Thirteen of these patients were not available for clinical evaluation but were assessed with radiography only (Fig 3). The patients were evaluated before surgery and at final follow-up. The mean observation time was 68 months. There was a minimum follow-up of 29 months (Table 1), and average age at surgery was 14.5 (SD = 1.0) (9-16) years. The average size of the tunnels used was 8.2 mm (range, 6-10).

**Table 1:** Characteristics of Included Patients

Patient characteristics	
Patients (n)	65
Gender, male	38 (58%)
Age at surgery (yr), mean (range)	14.5 (9-16)
Age at follow-up (yr), mean (range)	19.8 (17-23)
Follow-up time (mo)	68 (29-148)
Trauma mechanism	
Contact sport	51 (78.5%)
Noncontact sport	7 (10.8%)
Other	3 (4.6%)
Unknown	4 (6.2%)
Subjective outcome, baseline	
KOOS sport, median (IQR)	60.0 (25.0; 70.0)
KOOS QoL, median (IQR)	37.5 (25.0; 43.8)
Tegner, median (IQR)	3 (2; 4)

IQR, 25th and 75th interquartile range; SD, standard deviation.

### Femoral and Tibial Length

For femoral length a nonstatistically significant 1 mm difference between the operated and nonoperated sides was found. For tibia length a statistically significant 1 mm shortening of the operated tibia compared to the nonoperated side was found (Table 2). The total extremity length discrepancy was a nonsignificant 0.65 cm shortening of the operated extremity.

**Table 2:** Radiographic Length and Axis Difference Between the 2 Legs

	N = 65	*P* Values
Length difference (mm)		
Femur, mean (95% CI)	0.57 (−0.53; 1.67)	.30
Tibia, mean (95% CI)	−1.14 (−1.97; −0.30)	<.01∗
Total, mean (95% CI)	−0.65 (−2.21; 0.92)	.42
Angulation difference (^o^)		
Femur, mean (95% CI)	−1.47 (−2.26; −0.69)	<.01∗
Tibia, mean (95% CI)	−0.28 (−0.81; 0.25)	.30

CI, confidence interval; mMLFA, mechanical lateral distal femur angle; mMPTA, mechanical medial proximal tibial angle.

∗ Statistically significant difference (Student’s paired *t*-test). Results are presented as mean with 95% CI.

Six patients (9%) had more than 10 mm shortening of the operated leg. The most extreme difference measured was 21 mm shortening. This patient did not have measurable axis deviation difference (Table 3).

**Table 3:** Number of Patients With Radiographic Leg Length Shortening Compared to Uninjured Knee After Femoral Physis-Sparing ACL Reconstruction

Leg length difference	N = 65

>10 mm (range)	6 (9%) (11-21)
0 to ≤10 mm (range)	29 (45%) (0-10)
≤0 mm (range)	30 (46%) (−5 to 0)

### Axis Deviation

The average femoral-transcondylar angle at the operated side was found to be 1.51° of increased valgus compared to the nonoperated side, which was statistically different. The tibial-transcondylar angle at the operated side was found to be 0.25° of increased varus compared to the nonoperated side, which was not statistically different (Table 2). The number of patients with more than 5° of side-to-side difference was 6 (9%) all valgus deviation (Table 4). The most extreme valgus difference measured was 10° compared to the nonoperated side. None of the patients with leg length or valgus differences had symptoms that prompted surgical intervention. We found no relation between age and size of tunnels drilled with significant length or axis deviation differences.

**Table 4:** Number of Patients With More Than 5° Increased Valgus Compared to Opposite Knee After Femoral Physis-Sparing ACL Reconstruction

Knee angulation difference	N = 65

> 5^o^ (range)	6 (9%) (6-10)
0^o^ to ≤ 5^o^	41 (91%) (0-5)
≤ 0^o^ (range)	18 (−4 to 0)

### Clinical Outcome

Sixty-five patients were included in the study. Thirteen of these patients were not available for direct clinical examination. The side-to-side difference in knee laxity at follow-up was 2.4 (1.9-2.9) mm. KOOS Sport and KOOS QoL scores were 80 (62.5-87.5) and 75 (50.0-87.5). The IKDC score was 86.2 (62.1-95.4) and Tegner score was 5 (4-7) (Table 5).

**Table 5:** Clinical Outcome at Follow-Up After Femoral Physis-Sparing ACL Reconstruction of the Total Group

Knee Laxity	N = 52
Clinical outcome	
Difference in side-to-side laxity (mm), mean (CI)	2.40 (1.95; 2.86)
Subjective outcome	
KOOS sport, median (IQR)	80.0 (62.5; 90.0)
KOOS QoL, median (IQR)	75.0 (50.0; 87.5)
IKDC, median (IQR)	86.2 (62.1; 95.4)
Tegner, median (IQR)	5 (4; 7)

ACL, anterior cruciate ligament; CI, 95% confidence interval; IKDC, International Knee Documentation Committee subjective knee form; IQR, 25th and 75 interquartile range; KOOS, Knee Osteoarthritis Outcome Score; QoL, quality of life.

The KOOS sport, KOOS QoL, IKDC, and Tegner were registered.

The percentage of patients reaching MCID thresholds was 52% for KOOS Sport and 69% for KOOS QoL. The percentage of patients above PASS thresholds was 67% for both KOOS Sport and KOOS, the PASS for IKDC was 63%, and the MCID for Tegner was 79% (Table 6).

**Table 6:** Patients Meeting the MCID and PASS Thresholds at Follow-Up After Femoral Physis-Sparing ACL Reconstruction

	MCID (n = 29)	PASS (n = 52)
KOOS Sport	15 (52%)	35 (67%)
KOOS QoL	20 (69%)	34 (67%)
IKDC	—	32 (63%)
Tegner	23 (79%)	—

IKDC, International Knee Documentation Committee subjective knee form; KOOS, Knee Osteoarthritis Outcome Score; MCID, minimal clinically important difference; PASS, Patient Acceptable Symptom State; QoL, quality of life.

It was possible to calculate the MCID for 29 patients and PASS for all 52 patients.

Ten patients had an ACL graft rupture (15.4%), of which 8 patients had a revision ALCR. Three patients had a contralateral ACL rupture (4.6%) (Table 7). Six of the 13 patients with either ipsilateral graft rupture or contralateral ACL rupture had been involved in either soccer or team handball earlier than 12 months after surgery.

**Table 7:** Ipsilateral Rerupture Was Seen in 10 Patients and in the Contralateral Knee in 3 Cases

	N = 65
None	52 (80.0%)
Rerupture	13 (20%)
Ipsilateral	10
Contralateral	3
ALC Revision ipsilateral	8
ALC Revision contralateral	3
Nontreated rerupture	2 (3.1%)

ACL, anterior cruciate ligament

Eight of the 10 had a revision ACL reconstruction, and all 3 contralateral ACL ruptures were reconstructed.

## Discussion

The primary finding of the present study was that ALCR in skeletally immature patients using a femoral growth plate-sparing technique resulted in 9% limb length discrepancy and 9% develop valgus malformity. Physis injury during ALCR without transphyseal drilling has been suggested to occur by thermal influence because of the proximity of the tunnel drilling,^7^ and this is also described in an animal laboratory study.^14^^,^^18^

In the proximal tibia no angular deformity after surgery, despite the transphyseal drilling, was found. In a previous study, occurrence of tibial angular malformity after pediatric ALCR using transphyseal drilling has been described.^8^ In the previous study a transtibial femoral tunnel was drilled. The tibial tunnel was oblique and therefore crossing the physis in a more peripheral point. The more oblique tibial tunnel creates a larger oval-shaped and more peripherally-placed growth plate injury. In the present surgical technique where retro-drilling is used, the tibial tunnel could be placed independently of the intended femoral tunnel placement, and a more vertical tibial tunnel and more centrally placed tunnel is possible. This is in accordance with the finding in laboratory study with sheep by Seil et al.^19^ This might explain why no angular malformation is seen in the present study.

There are still conflicting results regarding growth disturbances after ALCR in skeletal immature patients. The rate of growth complication has been reported to be from 0 to 24 percent depending on the surgical technique used.^8^^,^^20^, ^21^, ^22^, ^23^

Wilson et al. ^24^ found no measurable difference after hybrid physeal sparing technique using the same technique as in this study. In a systematic review by Pierce et al.,^9^ no difference between transepiphyseal and epiphyseal drilling with respect to growth disturbances was found. The different findings could be explained by different diagnostic measures.

Limb length inequality of 0.5 to 1.5 cm is found in 32% of the adult population, and 4% have 1.5 cm limb length difference and is often not symptomatic.^25^

In the present study 9% with length growth abnormity with an overweight of minor limb shortening of the operated limb was found, with only one case of relative lengthening. This is in contrast to other results, where lengthening of the operated limb has been described.^21^^,^^26^ Interestingly, in a review by Collins et al.,^27^ more leg shortening was seen after transphyseal drilling, whereas leg lengthening was seen after an epiphyseal-sparing technique was used.

In a meta-analysis by Wong et al.^10^ with 1321 patients 4.4% of the patients developed growth disturbances of which a third required corrective surgery. Minor leg length difference has not been found to cause gait change.^28^ A notable gait asymmetry is seen only when the leg length difference exceeds 2 cm.^29^ In this study only 1 patient exceeded 2 cm leg length difference.

In the present study, growth disturbances were measured as leg length, and valgus-varus differences were registered. Growth disturbance in the sagittal plane was not measured. For ALCR the tunnels, as seen in the sagittal plane, are placed asymmetrically. They are placed in the anterior part of the tibial growth plate and in the posterior part of the femoral growth plate. Therefore one could expect a possible impact on growth in the sagittal plane.

The patients in the present study demonstrated overall a good clinical outcome with the present ALCR technique, based on knee stability, knee function, and subjective outcome scores. The measurement of side-to-side difference is from our experience with a high interexaminer variation. We found 2.4 mm side-to-side difference at follow-up as measured by 1 experienced physiotherapist. The finding was higher than presented in previous studies.^8^^,^^21^ However, in most of the studies on pediatric ALCR, instrumented measurements of knee laxity are not used at follow-up. In comparison to a recent Swedish study with evaluation of pediatric ALCR by Hansson et al.,^30^ this study presents comparable postoperative KOOS scores.

The incidence of graft rupture of 15% in the present study cohort of pediatric patients was comparable to failure rates reported in recent literature. Ho et al.^31^ found a graft failure rate of 17%. With a longer follow-up of 5 years, Fourman et al.^32^ reported a graft failure rate of 10.5%, Hansson et al.^30^ found a revision rate of 12%, and Sasaki et al.^33^ reported a graft rupture rate of 16.7 to 23.8%, depending on the surgical technique.

The clinical significance of the present study is the findings of no significant limb length disturbance and only minor and clinically nonsignificant valgus coronal plane disturbance after a hybrid epiphysis sparing ALCR. Also, the technique results in acceptable knee stability and subjective outcomes. Therefore the study indicates that ALCR in immature patients can be performed with only a small risk of growth disturbances and can be recommended for skeletally immature patients with ACL injury.

### Limitations

In this study we did not examine the growth changes in the sagittal plane. Any influence on growth in the sagittal plane therefore could not be detected. Also, we did not have a preoperative determination of remaining growth, which could give a more nuanced estimate of growth disturbance. However, at the time of surgery all included patients had open physis in both the distal femur and the proximal tibia as seen at radiography. Because this study describes a single surgeon series, the results may not be generalizable to other surgeons or institutions.

## Conclusion

The present femoral physis-sparing ALCR technique is associated with a low risk of alignment and length disturbance. The technique provides otherwise good subjective clinical outcomes and knee stability.

## Supplementary Data

ICMJE author disclosure forms
